# A comparison of postoperative morphometric and hemodynamic changes between saphenous vein and left internal mammary artery grafts

**DOI:** 10.14814/phy2.13487

**Published:** 2017-11-09

**Authors:** Tingting Fan, Yundi Feng, Feng Feng, Zhongjie Yin, Dayou Luo, Yuan Lu, Yingjin Xu, Wenchang Tan, Yunlong Huo

**Affiliations:** ^1^ Mechanics and Engineering Science College of Engineering Peking University Beijing China; ^2^ Department of Cardiology Affiliated Hospital of Xuzhou Medical University Xuzhou China; ^3^ Department of Radiology Affiliated Hospital of Hebei University Hebei University Baoding China; ^4^ PKU‐HKUST Shenzhen‐Hongkong Institution Shenzhen China; ^5^ Shenzhen Graduate School Peking University Shenzhen China

**Keywords:** CABG, flow simulation, hemodynamics, LIMA, SVG

## Abstract

There is higher long‐term failure of the saphenous vein graft (SVG) compared with the left internal mammary artery (LIMA) graft, which is affected by the hemodynamic environment. A comprehensive analysis of postoperative structure‐function changes is important to study the atherogenesis in the SVG. A comparison of morphometric and hemodynamic parameters was carried out between LIMA grafts and SVGs and between different time points postoperatively. Various parameters were obtained from the image reconstruction and flow simulation in patients, who underwent CT exams for ~1 year, 5 and 10 years after revascularization. Morphometric data showed a decrease in lumen size in the entire SVG and anastomosis of different patients in a sequence of ~1 year, 5 and 10 years postoperatively despite negligible changes of LIMA size. Computational results indicated the fourfold increased surface area ratio (SAR) of low time‐averaged wall shear stress (TAWSS) in the SVG and anastomosis at postoperative 10 years than that at postoperative ~1 year. The SAR of high TAWSS gradient (TAWSSG) at the distal anastomosis between SVG and coronary arteries was significantly higher (14 ± 9% vs. 6 ± 8%) than that in the LIMA group at postoperative ~1 year. There were strong correlations between morphometric and hemodynamic parameters in the SVG and distal anastomosis at various time points postoperatively, which showed deterioration relevant to persistent diffuse diseases at postoperative ~10 years.

## Introduction

Coronary artery bypass graft (CABG) can effectively relieve symptoms of ischemic heart diseases (Hillis et al. 2011). The left internal mammary artery (LIMA) graft and saphenous vein graft (SVG) were the most frequently used CABGs because of the low failure rate and easy operability, respectively (Campeau et al. 1983; Loop et al. 1986; Fujiwara et al. 1988; Motwani and Topol 1998; Goldman et al. 2004; Parang and Arora 2009). In comparison with the LIMA graft, the long‐term SVG patency was not completely satisfied given multiple hemodynamic risk factors, for example, low time‐averaged wall shear stress over a cardiac cycle (low TAWSS), high oscillatory shear index (high OSI) and high TAWSS gradient (high TAWSSG) (Berger et al. 2004; Goldman et al. 2004). As previous hemodynamic studies were mainly associated with the intimal hyperplasia that generally occurs within ~1 year after revascularization (Kassab and Navia 2006; Loth et al. 2008; Ghista and Kabinejadian 2013), we have recently carried out the hemodynamic analysis to investigate the long‐term patency of SVG (Huo et al. 2013) and LIMA graft (Fan et al. 2016). To the best of our knowledge, there is lack of a comprehensive comparison of morphometry and hemodynamics between SVGs and LIMA grafts and between different time points postoperatively. The comparative study can enhance our understanding of the hemodynamic mechanisms of SVG failure and hence reduce the incidence of repeat revascularization.

Hemodynamic parameters, for example, low WSS, high OSI and WSSG, are related to stagnation, reversal and vortical flows (Kleinstreuer et al. 2001; Huo et al. 2007), which result in abnormal biological responses such as dysfunction of endothelial cells, monocyte deposition, elevated wall permeability to macromolecules, particle migration into the vessel wall, smooth muscle cell proliferation, microemboli formation, and so on (Chiu and Chien 2011). Malek et al. (1999) showed a threshold TAWSS ≤ 4 dynes/cm^2^; Nordgaard et al. (2010) indicated a threshold OSI ≥ 0.15; and Fan et al. (2016) proposed a threshold TAWSSG ≥ 500 dynes/cm^3^ for the incidence and progression of atherosclerosis. The growth of atheroma leads to the complex distribution of hemodynamic parameters in the cardiovascular system (Huo et al. 2007, 2009). Given the pulsatility of blood flow, the sites of stagnation, reversal and vortical flows can moderately change over a cardiac cycle. The surface area ratios of low TAWSS (SAR‐TAWSS), high OSI (SAR‐OSI) and TAWSSG (SAR‐TAWSSG) were proposed to accurately feature the disturbed flow patterns in the graft and coronary arteries (Fan et al. 2016).

The objective of this study is to perform the morphometric and hemodynamic analysis in patient‐specific SVGs and LIMA grafts for ~1 year, 5 and 10 years after surgical revascularization. We hypothesize a severe deterioration of hemodynamic parameters (e.g., SAR‐TAWSS and SAR‐OSI) in SVGs and anastomoses of different patients in a sequence of postoperative ~1 year, 5 and 10 years despite relatively small difference in LIMA grafts. The correlation between the highest SAR‐TAWSS and the smallest lumen size of SVGs and anastomoses in patients at postoperative ~10 years indicated the high incidence of mild atherosclerotic lesions as compared with those at postoperative ~1 year and 5 years. To test the hypotheses, the geometry of the graft and coronary arteries was reconstructed from CTA images of 132 patients. We computed multiple hemodynamic parameters, that is, TAWSS, OSI, TAWSSG and transWSS (transverse WSS) (Mohamied et al. 2015) as well as SAR‐TAWSS, SAR‐OSI, SAR‐TAWSSG and SAR‐transWSS in the graft and at the anastomosis between the graft and coronary artery (see the detailed definitions in the Nomenclature).

## Methods

### Study design

**Figure 1:**
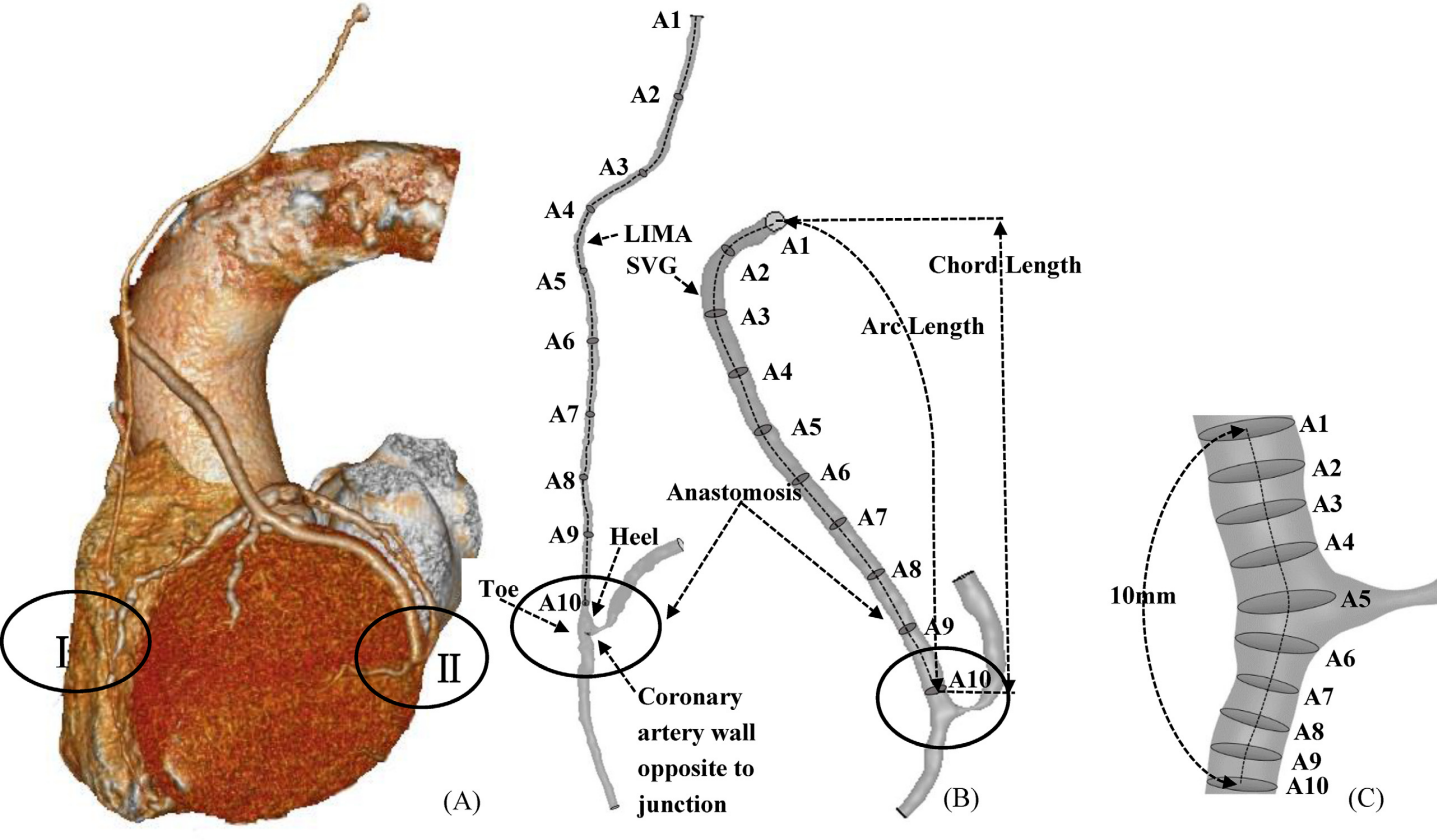
(A) 3‐dimensional (3D) geometric model of LIMA graft and SVG and coronary arteries reconstructed from CTA images of a representative patient; (B) schematic representative of geometric parameters in LIMA graft and SVG, and (C) schematic representative of geometric parameters at an anastomosis.

As shown in Figure 1A, we retrospectively analyzed two groups of 132 patients with end‐to‐side anastomoses and severe coronary stenoses, who underwent LIMA grafting to the left anterior descending, LAD, artery (i.e., the LIMA group including 69 patients), or SVG connecting to the left circumflex, LCx, artery, LAD, or primary branches of the epicardial LAD arterial tree (i.e., the SVG group including 63 patients). These patients underwent CTA exams of graft and coronary arteries at ~1 year (22 patients in the LIMA group and 21 patients in the SVG group), ~5 years (25 patients in the LIMA group and 21 patients in the SVG group), and ~10 years (22 patients in the LIMA group and 21 patients in the SVG group) after revascularization. None of the 132 patients were studied longitudinally.

The study was approved by the Institutional Review Board (IRB) for the Affiliated Hospital of Xuzhou Medical University, which confirms the declaration of Helsinki. All patients gave the signed informed consent for all methods of the study performed in accordance with the relevant guidelines and regulations of the IRB for the Affiliated Hospital of Xuzhou Medical University.

### Image acquisition

Similar to previous studies (Huo et al. 2013; Fan et al. 2016), CTA scans of patients were conducted on a dual‐source CT scanner (Siemens Definition, Forchheim, Germany) when the heart rate was ≤ 65 bpm (beats per minute). After an initial survey scan, CTA images were acquired when contrast agent (Iopromide‐Ultravist 370, Bayer Healthcare, Morristown) at the dose of 1.0 mL/kg was injected at a rate of 5 mL/s followed by IV injection of 50 ml saline at a rate of 5 ml/s. Study parameters included the following: 2 × 64 × 0.6 mm collimation, tube voltage – 120 kV; tube current – adjusted to body size; gantry rotation time – 330 msec; pitch – 0.2–0.43 depending on heart rate. The simultaneous acquisition of multi‐parallel cross sections enabled the imaging of the bypass graft and coronary arteries in a single breath hold. Images were reconstructed with a slice thickness/increment of 0.7/0.4 mm with B26f at temporal resolution of 83 msec (half‐scan). The initial data window was positioned at 70% of the R‐R interval, with additional data sets reconstructed at ±5% intervals to compensate for motion artifacts in coronary arteries as needed.

### Geometrical model

As shown in Figure 1B, morphometric data of the bypass graft and coronary arteries were extracted from patient CTA images using the MIMICS software (Materialise, NV, Belgium). A centerline was formed by a series of center points which was located in the center on the cross–sectional views of the contour of the 3D vessel. Subsequently, the best fit diameter, D_fit_, was calculated as twice the average radius between the point on the centerline and the contour forming the contour of the 3D vessel.

The CTA reconstruction and imaging analysis were performed by researchers (Tingting Fan, Feng Feng, Dayou Luo and Yundi Feng) at the College of Engineering, Peking University as well as a Radiologist (Yingjin Xu) at the Affiliated Hospital of Hebei University and a Cardiologist (Yuan Lu) at the Affiliated Hospital of Xuzhou Medical University. The reproducibility of those measurements showed *κ* value equal to 0.87 (Cohen 1986).

Based on these morphometric data, geometrical models were meshed using the ANSYS ICEM software (ANSYS Inc., Canonsburg) and then smoothed using the Geomagic Studio software (3D Systems, Rock Hill). Similar to a previous study (Yin et al. 2016), a mesh dependency was conducted such that the relative error in two consecutive mesh refinements was < 1% for TAWSS and OSI. A total of approximately 500,000‐600,000 tetrahedral shaped volume elements (element size = 0.29 mm) with finer meshes near the vessel wall were necessary to accurately mesh the computational domain.

### Computational model

The Navier‐Stokes and continuity equations (see details in the Appendix) were solved using a finite volume solver, FLUENT (ANSYS Inc., Canonsburg). The bypass grafts and coronary arteries were assumed to be rigid and impermeable. Three cardiac cycles were required to achieve the convergence for the transient analysis similar to previous studies (Huo et al. 2007, 2009, 2012, 2013). A constant time step was employed, where ∆*t* = 0.01 sec with 84 total time steps per cardiac cycle. The aortic pressure wave of a patient measured in a previous study (Fan et al. 2016) was normalized by the time‐averaged value and then scaled back to the physiological range based on the systolic and diastolic pressures measured in each patient. The scaled pressure wave was set as the boundary condition at the inlet of the graft and coronary artery. The resistance boundary condition was assigned to each outlet (see details in the Appendix). Although the blood is a suspension of particles, it behaves as a Newtonian fluid in vessels with diameters > 1 mm (Nichols and McDonald 2011). Moreover, we have recently shown negligible difference of hemodynamic parameters between Newtonian and non‐Newtonian (i.e., Carreau fluid) models (Yin et al. 2016). Hence, the viscosity (*μ*) and density (*ρ*) of the solution were set as 4.5 × 10^−3^ Pa∙s and 1060 kg/m^3^, respectively, to mimic blood flows with a hematocrit of about 45% in these arteries. Hemodynamic parameters including the WSS, OSI, WSSG, and transWSS (see details in the Appendix) were determined from the computed flow fields. Furthermore, we computed the SAR‐TAWSS, SAR‐OSI, SAR‐TAWSSG, and SAR‐transWSS in the graft and at the anastomosis between the graft and coronary artery. SAR‐TAWSS, SAR‐OSI, SAR‐TAWSSG, and SAR‐transWSS represent the surface area ratios of low TAWSS (TAWSS ≤ 4 dynes/cm^2^) and high OSI (OSI ≥ 0.15), TAWSSG (TAWSSG ≥ 500 dynes/cm^3^), and transWSS (transWSS ≥ 6 dynes/cm^2^), respectively, in the graft or distal anastomosis.

### Statistical Analysis

The mean±SD (standard deviation) values of morphometric and hemodynamic parameters were computed by averaging over all subjects in a population. The best fit diameter, D_fit_, was plotted along the graft centerline, which was normalized by the accumulative length from the center of A1 to A10 in Figure 1B. The best fit diameter, D_fit_, was also plotted along the anastomotic centerline, which was normalized by the accumulative length from the center of A1 to A10 in Figure 1C. A two‐way ANOVA was used to detect the statistical difference of these parameters between LIMA and SVG groups and between different time points postoperatively. A *P*‐value < 0.05 was indicative of a significant difference between two populations.

## Results

Table 1 lists the demographics of the study population in LIMA and SVG groups. Patients in each group were divided into three subgroups based on the CTA scanning time at ~1 year, 5 and 10 years postoperatively. All patients had severe coronary stenoses (i.e., area stenosis>80% in Table 2). Most patients were hypertensive before revascularization and still had high systolic blood pressures when they underwent CTA scans. The two groups showed similar values (no statistical difference) of fasting glucose, cholesterol, triglycerides, LDL, and HDL.

**Table 1:** Demographics of the study population in LIMA grafts and SVGs at different postoperative times

Parameters	LIMA group			SVG group		
Postoperative time (y)	~1	~5	~10	~1	~5	~10
Patient number	22	25	22	21	21	21
Age (y)	68 ± 7	65 ± 7	72 ± 6	67 ± 8	68 ± 6	70 ± 7
Male (%)	86	76	77	86	57	76
BMI, kg/m^2^	24 ± 1.5	24 ± 2.6	25 ± 2.0	25 ± 2.2	24 ± 2.5	25 ± 1.9
Demographics of the study population before surgical revascularization						
Hypertension (%)	78	82	72	68	91	65
Hypercholesterolemia (%)	59	59	26	37	32	27
Diabetes mellitus (%)	39	43	63	47	27	40
Myocardial infarction (%)	24	10	37	18	30	41
Smoking (%)	56	38	68	67	36	42
Demographics of the study population before CTA scanning						
Systolic blood pressure (mmHg)	149 ± 19	148 ± 17	155 ± 19	147 ± 19	162 ± 19	150 ± 19
Diastolic blood pressure (mmHg)	87 ± 11	82 ± 14	88 ± 10	82 ± 9.0	72 ± 15	87 ± 12
Fasting glucose (mmol/l)	5.9 ± 1.6	6.5 ± 1.6	6.0 ± 1.7	7.2 ± 2.7	6.1 ± 1.7	6.0 ± 1.4
Cholesterol (mmol/l)	4.7 ± 0.7	4.3 ± 1.1	4.6 ± 1.1	4.5 ± 1.0	4.3 ± 1.1	4.3 ± 1.1
Triglycerides (mmol/l)	1.5 ± 0.9	1.3 ± 0.6	1.6 ± 1.4	1.3 ± 0.6	1.5 ± 0.7	1.5 ± 1.3
LDL (mmol/l)	2.5 ± 0.9	2.2 ± 1.2	2.0 ± 1.2	2.5 ± 0.7	2.2 ± 1.8	2.1 ± 1.8
HDL (mmol/l)	1.3 ± 0.4	1.4 ± 0.4	1.2 ± 1.0	1.2 ± 0.4	1.4 ± 0.4	1.5 ± 0.6

**Table 2:** Morphometric and hemodynamic parameters in patients of LIMA and SVG groups

Postoperative time (y)	~1		~5		~10	
Vessel Groups	LIMA	SVG	LIMA	SVG	LIMA	SVG
Morphometric parameters						
D_inlet_	2.3 ± 0.6	3.3 ± 0.9	2.5 ± 0.7	2.7 ± 0.7	2.4 ± 0.8	2.6 ± 0.8
D_mean_	3.2 ± 0.4	4.5 ± 0.6	3.2 ± 0.4	4.1 ± 0.6	3.1 ± 0.5	3.7 ± 0.7
$\frac{L_{\mathrm{chord}}}{L_{\mathrm{arc}}}$	0.9 ± 0.1	0.9 ± 0.1	0.9 ± 0.1	0.8 ± 0.1	0.9 ± 0.1	0.8 ± 0.1
D_anas_	2.4 ± 0.7	4.0 ± 0.8	2.5 ± 0.6	3.4 ± 0.6	2.5 ± 0.6	3.1 ± 0.7
Coronary area stenosis (%)	85 ± 2	84 ± 8	86 ± 5	87 ± 5	90 ± 3	89 ± 7

		~1 y versus ~5 y		~5 y versus ~10 y		~10 y versus ~1 y	
A comparison of hemodynamic parameters in the graft between different postoperative time							
*P*‐value (time vs. time)	SAR‐TAWSS	0.42	<0.05	0.44	<0.05	0.15	<0.05
	SAR‐OSI	0.63	0.78	0.77	0.07	0.40	0.05
A comparison of hemodynamic parameters in the anastomosis between different postoperative time							
*P*‐value (time vs. time)	SAR‐TAWSS	<0.05	<0.05	0.14	<0.05	<0.05	<0.05
	SAR‐OSI	0.69	0.99	0.33	0.78	0.36	0.48
	SAR‐TAWSSG	0.63	<0.05	0.07	0.09	<0.05	0.95

**Figure 2:**
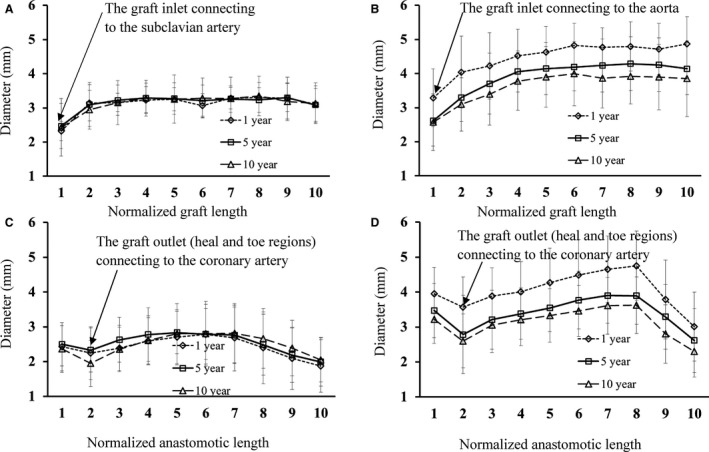
(A–B) plot of D_fit_ along normalized graft centerline in LIMA (A: *n* = 22 for ~1 year, *n* = 25 for ~5 years, and *n* = 22 for ~10 years postoperatively) and SVG (B: *n* = 21 for ~1 year, *n* = 21 for ~5 years, and *n* = 21 for ~10 years postoperatively) groups; (C‐D) plot of D_fit_ along normalized anastomotic centerline in LIMA (C: *n* = 22 for ~1 year, *n* = 25 for ~5 years, and *n* = 22 for ~10 years postoperatively) and SVG (D: *n* = 21 for ~1 year, *n* = 21 for ~5 years, and *n* = 21 for ~10 years postoperatively) groups, where error bars refer to the SDs of D_fit_ at various postoperative times. The centerlines were normalized by the accumulative length from A1 to A10 in Figure 1B and C.

Figure 2A and B plot D_fit_ along the normalized graft centerline in LIMA and SVG groups, respectively, while Figure 2C and D show D_fit_ along the normalized anastomotic centerline. The centerlines are normalized by the accumulative length from A1 to A10 in Figure 1B and C, respectively. Patients in the LIMA group show negligible changes of diameters within postoperative ~10 years. However, the SVGs of different patients in a sequence of postoperative ~1 year, 5 and 10 years have a decrease in diameters, which show significant difference between different time points. Patients of LIMA and SVG groups have similar diameters for ~10 years after revascularization. Grafts have the minimal diameters at the inlet and outlet, as shown in Figure 2. Table 2 lists the mean ± SD values of morphometric parameters in LIMA and SVG groups, where D_inlet_, D_mean_, L_chord_, L_arc_, and D_anas_ refer to the inlet diameter of a graft, the averaged diameter in the entire graft except for the anastomotic region, the accumulative length along the entire graft, the straight length from the inlet to the distal anastomosis of the graft, and the averaged diameter in the anastomosis, respectively. Morphometric parameters, D_inlet_, D_mean_, and D_anas_, decrease with time in the SVG group, but remain relatively unchanged in the LIMA group. The ratios of L_chord_ to L_arc_ in the LIMA group are similar to those in the SVG group.

**Figure 3:**
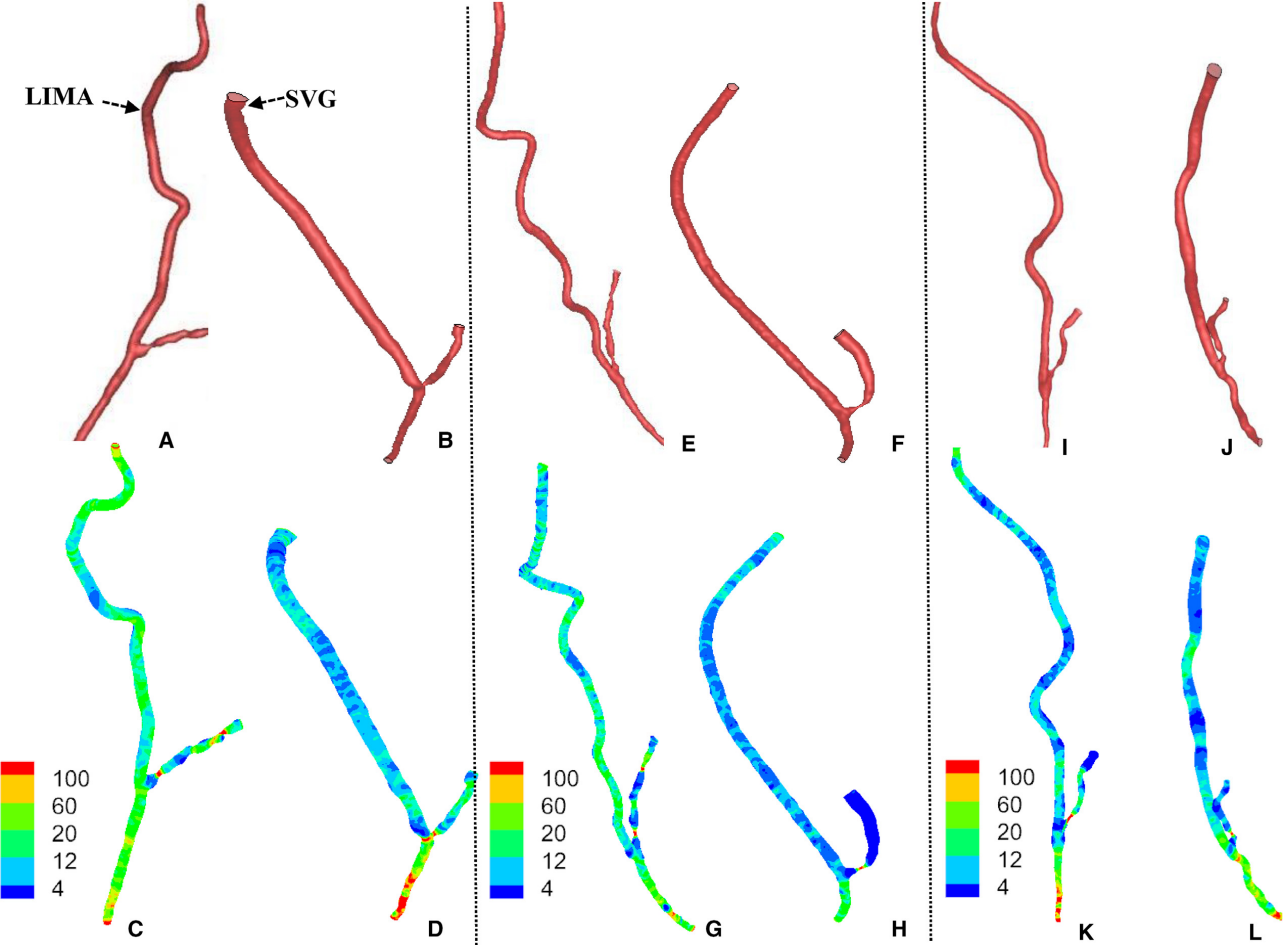
(A–D) 3D geometric model of LIMA graft (A) and SVG (B) and TAWSS (Unit: Dynes∙cm^−2^) in LIMA graft (C) and SVG (D) of two representative patients, who underwent CTA for ~1 year after surgical revascularization; (E–H) 3D geometric model and TAWSS (Unit: Dynes∙cm^−2^) in LIMA graft and SVG of two representative patients, who underwent CTA for ~5 year after surgical revascularization; (I–L) 3D geometric model and TAWSS (Unit: Dynes∙cm^−2^) in LIMA graft and SVG of two representative patients, who underwent CTA for ~10 year after surgical revascularization.

**Figure 4:**
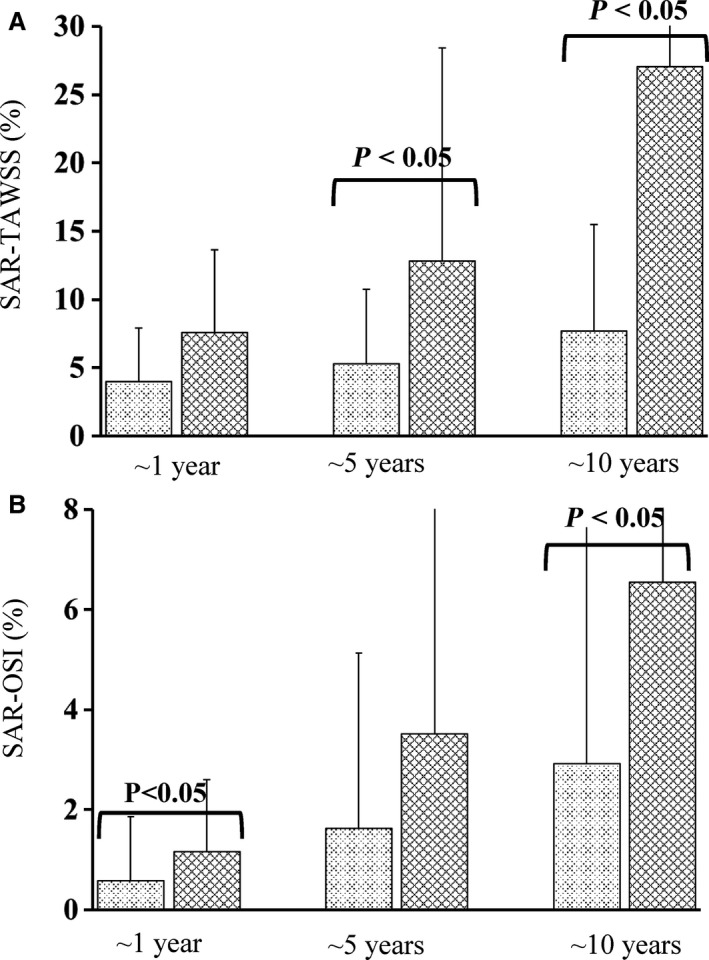
(A) SAR‐TAWSS and (B) SAR‐OSI (*n* = 22 for ~1 year, *n* = 25 for ~5 years, and *n* = 22 for ~10 years postoperatively in the LIMA group; and *n* = 21 for ~1 year, *n* = 21 for ~5 years, and *n* = 21 for ~10 years postoperatively in the SVG group) in the entire graft except for the anastomotic regions. The histograms of light and dark textures refer to the mean values of the parameters averaged over patients in LIMA and SVG groups, respectively, at various postoperative times, where error bars refer to the SDs of the parameters and *P*‐value < 0.05 indicates statistical difference between LIMA and SVG groups.

**Figure 5:**
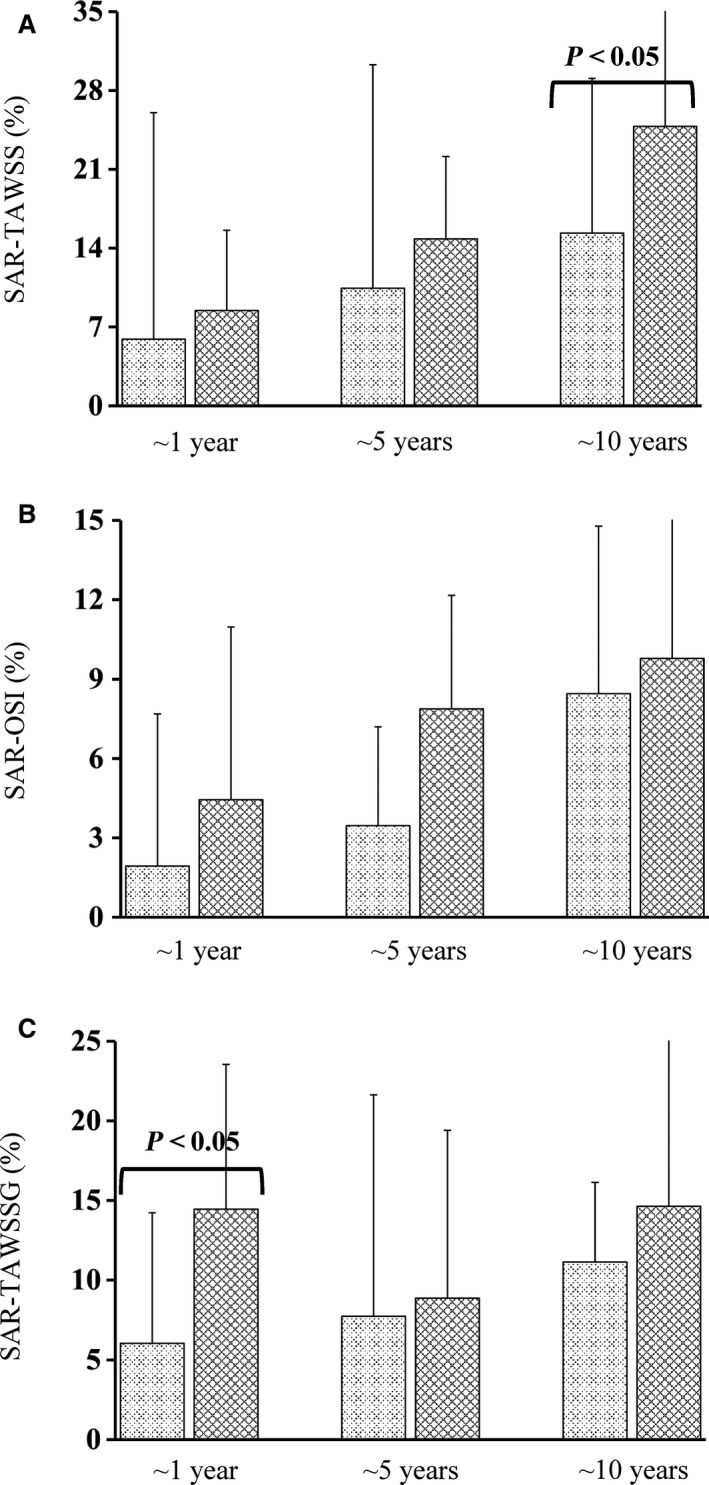
(A) SAR‐TAWSS, (B) SAR‐OSI, and (C) SAR‐TAWSSG (*n* = 22 for ~1 year, *n* = 25 for ~5 years, and *n* = 22 for ~10 years postoperatively in the LIMA group; and *n* = 21 for ~1 year, *n* = 21 for ~5 years, and *n* = 21 for ~10 years postoperatively in the SVG group) at the anastomosis between the graft and coronary arteries. The histograms of light and dark textures refer to the mean values of the parameters averaged over patients in LIMA and SVG groups, respectively, at various postoperative times, where error bars refer to the SDs of the parameters and *P*‐value < 0.05 indicates statistical difference between LIMA and SVG groups.

Figure 3 shows the 3D geometry and the TAWSS distribution in representative SVGs and LIMA grafts at ~1 year, 5 and 10 years postoperatively. Figure 4A and B show the mean±SD values of SAR‐TAWSS and SAR‐OSI in the graft as well as a comparison of the two parameters between SVGs and LIMA grafts at different time points. Accordingly, Figure 5A–C show the values and comparison of SAR‐TAWSS, SAR‐OSI, and SAR‐TAWSSG at the anastomosis between the graft and coronary arteries. Moreover, Table 2 lists a comparison of these parameters between different time points. Since SAR‐TAWSSG values are < 1% in the graft except for the anastomotic region and SAR‐transWSS values are < 0.3% in the graft and anastomosis, they are not used for further analysis. There is an increase in SAR‐TAWSS and SAR‐OSI in the graft and anastomosis of different patients for a sequence of ~1 year, 5 and 10 years after revascularization. Table 2 shows statistical difference of SAR‐TAWSS in the SVG and anastomosis and SAR‐TAWSS and SAR‐TAWSSG at the anastomosis of LIMA patients between 1 year and 10 years. Moreover, as shown in Figures 4 and 5, at postoperative ~1 year, SAR‐OSI in the SVG and SAR‐TAWSSG at the anastomosis between SVG and coronary artery are significantly higher (≥ twofold) than those in the LIMA group; at postoperative ~5 years, SAR‐TAWSS in the SVG are 2.5 times higher (*P*‐value < 0.05) than those in the LIMA; at postoperative ~10 years, SAR‐TAWSS in the SVG and anastomosis are 1.5‐3.5 times higher (*P*‐value < 0.05) and SAR‐OSI in the SVG is 2.2 times higher (*P*‐value < 0.05), compared with the LIMA group.

## Discussion

We carried out the morphometric and hemodynamic analysis in the graft (LIMA vs. SVG) and coronary arteries with the end‐to‐side anastomosis as well as severe coronary stenoses, reconstructed from CTA images of 132 patients. Three major findings are reported as: (1) Patients at postoperative ~1 year have the maximal diameters in the SVG and anastomosis while patients at postoperative ~10 years have the minimal values (*P*‐value < 0.05); (2) There is relatively small difference of diameters in the LIMA group over time while patients in SVG and LIMA groups have similar diameters for ~10 years after revascularization; (3) SAR‐TAWSS and SAR‐OSI increase in the graft and anastomosis of different patients for a sequence of postoperative ~1 year, 5 and 10 years, which shows significant difference of SAR‐TAWSS in the SVG and anastomosis between different time points, but no statistical difference of SAR‐OSI; and (4) SAR‐TAWSSG in the anastomosis at postoperative ~1 year and SAR‐TAWSS in the graft and anastomosis at postoperative ~10 years are significantly higher in the SVG group than those in the LIMA group. The findings are discussed in relation to the potential improvement of graft patency.

The LIMA is generally considered as the first choice for grafting to the LAD while the SV is selected as a supplementary graft given its ease of use (Canver 1995). Patency at 10 years was ~58% for SVGs compared with ~85% for LIMA grafts (Goldman et al. 2004). Biomechanical stresses are important for the vascular remodeling and hence result in partial or total occlusion of the vessel lumen (Kassab and Navia 2006). Although a sudden and significant increase in intramural stresses and strains contributes to the thrombosis and intimal hyperplasia (IH) within the initial years (~1 year in general) after grafting (Hozumi et al. 1996), abnormal hemodynamic parameters (e.g., low TAWSS, high OSI and TAWSSG) lead to the development of atherosclerosis in the arterialized SVG after the initial years of surgical revascularization (Motwani and Topol 1998). This study investigated the effects of hemodynamics on the long‐term graft patency. Moreover, a group of hemodynamic parameters (i.e., SAR‐TAWSS, SAR‐OSI, SAR‐TAWSSG, and SAR‐transWSS), defined in a previous study (Fan et al. 2016), was used to quantify the complex distribution of low TAWSS and high OSI, TAWSSG and transWSS in the graft and anastomosis. SAR‐transWSS values are < 0.2% in the graft and anastomosis and hence neglected here. This parameter may be better for the hemodynamic analysis in the large peripheral arteries (Peiffer et al. 2013; Mohamied et al. 2015).

### Changes in the graft except for the anastomotic Region

Although arterialized SVGs had larger diameters than LIMA grafts in patients at postoperative ~1 year, they had similar diameters (no statistical difference between SVG and LIMA groups) in patients for ~10 years after revascularization (Fig. 2A vs. B). The diameters remained relatively constant in LIMA grafts within postoperative ~10 years. Corresponding to the morphometric changes, there was a significant increase in SAR‐TAWSS in the SVG of different patients in a sequence of postoperative ~1 year, 5 and 10 years despite a slight increase in the LIMA graft in Table 2. The recurrence of atherosclerotic lesions is the most problematic risk factor for long‐term SVG failure owing to proliferation and migration of medial smooth muscle cells (SMC) into the neointima and significant deposition of interstitial collagen and extracellular matrix (Schwartz et al. 1995; Motwani and Topol 1998). The sites of low TAWSS (e.g., proximal/distal sites to a mild stenosis) are predisposed to the atherosclerotic growth (Chiu and Chien 2011; Huo et al. 2013). The correlation between the highest SAR‐TAWSS and the minimal lumen diameters indicated the highest incidence of mild atherosclerotic lesions (i.e., persistent diffuse diseases) (Kalan and Roberts 1990; Motwani and Topol 1998) in the entire SVG of patients at postoperative ~10 years given no observed severe stenoses. On the other hand, the mechanical properties of the LIMA graft are nearly matched to those of the coronary artery that is being bypassed. Hence, the small changes of SAR‐TAWSS as well as the low values of SAR‐TAWSSG (< 1%) and SAR‐OSI (<3%) represented a slight perturbation to the LIMA graft, which minimized the remodeling response and approximately maintained the lumen size in the entire LIMA graft within postoperative ~10 years.

### Anastomotic changes

The anastomosis between the graft and coronary artery is the most frequent site for the graft failure (Fei et al. 1994; Hofer et al. 1996; Ghista and Kabinejadian 2013). In agreement with previous studies (Sottiurai et al. 1989; Bassiouny et al. 1992), there were severe stenoses at the toe and heel regions of the anastomosis, as shown in Figure 2C and D, owing to the significantly higher values of SAR‐TAWSS, SAR‐OSI and SAR‐TAWSSG at the anastomosis compared with other sites in the graft (Fig. 5 vs. Fig. 4). There was significant difference of SAR‐TAWSS at the SVG anastomosis between different time points, which showed similar changes over time to those in the graft. In comparison with the anastomosis between LIMA and coronary arteries, the SVG anastomosis had higher values of SAR‐TAWSSG at postoperative ~1 year, which could also lead to the vascular remodeling given similarity of other risk factors (i.e., hypertension, smoking and dyslipidemia) between LIMA and SVG groups. Hence, multiple hemodynamic parameters were associated with the anastomotic remodeling between SVG and coronary arteries at different time points.

There was a smaller lumen diameter of the graft near the bifurcation between LIMA and subclavian artery and between SVG and aorta compared with other sites in the graft except for the distal anastomotic region, as shown in Figure 2A and B. The small diameter near the bifurcation between LIMA and subclavian artery of old patients was due to the atherosclerotic development before surgical revascularization (Huo et al. 2007, 2009; Finet et al. 2010).

### Potential implications for graft patency

The comparison of morphometric and hemodynamic changes between patients of different time points implied that the vicious cycle of increased SAR‐TAWSS and decreased diameters could lead to the growth of diffuse diseases in the graft and anastomosis over time, the confirmation of which needs longitudinal follow up. Moreover, the significant increase in SAR‐TAWSS and SAR‐OSI in the SVG and distal anastomosis with time (SVG vs. LIMA and ~10 years vs. ~1 year) implies an enhanced graft attrition rate over 10 years after revascularization (Hillis et al. 2011), which requires long‐term clinical studies in a group of patients.

### Critique of model

Although this study showed the correlation between SAR‐TAWSS and lumen size, more invasive studies (e.g., IVUS or OCT exams) are also needed to find the relation between hemodynamic changes and chronic endothelial cell injury and dysfunction in the graft and distal anastomosis (Motwani and Topol 1998). Since the present analysis was not repeated over time, perspective studies are required to follow the morphometric and hemodynamic changes in patients postoperatively.

## Conclusions

The retrospective study showed morphometric and hemodynamic changes in LIMAs or SVGs of different patients for ~1 year, 5 and 10 years after surgical revascularization. There were strong correlations between high SAR‐TAWSS and small lumen size in the SVG and distal anastomosis at different time points despite the negligible changes in the LIMA graft. The high SAR‐TAWSSG in the anastomosis between SVG and coronary arteries could also contribute to the vascular remodeling within the initial years after grafting. The findings can improve our understanding of hemodynamic mechanisms for graft failure.

## Conflict of interest

The authors declare no conflicts of financial/personal interest.

## Computational Models

### 3‐D CFD Model

The governing equations are formulated for the bypass graft‐coronary artery, each vessel of which is assumed to be rigid and to have impermeable wall. The equations of continuity and Navier‐Stokes can be written as:

(A1) $$\nabla \cdot \vec{V}=0$$

(A2) $$\rho \frac{\partial \vec{V}}{\partial t}+\rho \vec{V}\cdot \nabla \vec{V}=-\nabla P+\nabla \cdot \mu \left(\nabla \vec{V}+{\left(\nabla \vec{V}\right)}^{T}\right)$$

where $\vec{V}=u{\hat{e}}_{x}+v{\hat{e}}_{y}+w{\hat{e}}_{z}$, *P*,* ρ*, and *μ* represent velocity, pressure, blood mass density, and viscosity, respectively.

### Resistance Boundary Condition

These patients were diagnosed with no microvascular diseases through the Duke Activity Status Index. The total coronary flow, $Q_{\mathrm{total}}$ (time‐averaged over a cardiac cycle), was estimated from CT images of myocardial mass using the scaling law (Choy and Kassab 2008). Based on the diameter‐flow scaling law (Huo and Kassab 2012), the coronary flow at outlet *i*, ${\left(Q_{\mathrm{mean}}\right)}_{i}$ (time‐averaged over a cardiac cycle), was estimated as:

(A3) $${\left(Q_{\mathrm{mean}}\right)}_{i}=Q_{\mathrm{total}}\times \frac{D_{i}^{7/3}}{\sum_{j=1}^{N}D_{j}^{7/3}}$$

(A4) $${\left(V_{\mathrm{mean}}\right)}_{i}=\frac{{\left(Q_{\mathrm{mean}}\right)}_{i}}{\frac{\pi }{4}\times D_{i}^{2}}$$

where *N* is the total number of outlets of coronary arterial trees reconstructed from CTA images and ${\left(V_{\mathrm{mean}}\right)}_{i}$ (time‐averaged over a cardiac cycle) is the outlet flow velocity.

A steady‐state flow simulation was carried out with the inlet pressure of $\left(P_{\mathrm{mean}}-P_{0}\right)$ and outlet flow velocity, where $P_{\mathrm{mean}}$ is the time‐averaged aortic pressure over a cardiac cycle and the zero‐flow pressure, $P_{0}$, was set to 51 mmHg (Dole et al. 1984). The coronary pressure at outlet *i*, ${\left(P_{\mathrm{mean}}\right)}_{i}$ (time‐averaged over a cardiac cycle), was obtained from the steady‐state computation. The resistance at each outlet of the epicardial coronary arterial tree, $R_{\mathrm{outlet}}$, can be determined as:

(A5) $$R_{\mathrm{outlet}}=\frac{{\left(P_{\mathrm{mean}}\right)}_{i}-P_{0}}{{\left(Q_{\mathrm{mean}}\right)}_{i}}$$

In the transient flow simulation, $R_{\mathrm{outlet}}$ is assumed to be constant. Hence, we obtained the following equation as:

(A6) $$V_{\mathrm{outlet}}=\frac{P_{\mathrm{outlet}}-P_{0}}{R_{\mathrm{outlet}}\times \frac{\pi }{4}\times D_{\mathrm{outlet}}^{2}}$$

where $P_{\mathrm{outlet}}$ and $V_{\mathrm{outlet}}$ refer to the transient pressure and flow velocity, respectively, at each outlet of the epicardial coronary arterial tree. Equation [A6] was used as the transient resistance boundary condition with $V_{\mathrm{outlet}}$ and $P_{\mathrm{outlet}}$ being the transient variables in a cardiac cycle.

### Hemodynamic Parameters

Similar to a previous study (Fan et al. 2016), at any point of 3‐D FVM, the stress can be represented as a nine‐component tensor ($\bar{\bar{\tau }}$), which can be written as follows:

(A7) $$\bar{\bar{\tau }}=\left[\begin{array}{ccc} \tau_{11} & \tau_{12} & \tau_{13}\\ \tau_{21} & \tau_{22} & \tau_{23}\\ \tau_{31} & \tau_{32} & \tau_{33} \end{array}\right]2\mu \bar{\bar{D}}=\mu \left[\begin{array}{ccc} 2\frac{\partial u}{\partial x} & \frac{\partial u}{\partial y}+\frac{\partial v}{\partial x} & \frac{\partial u}{\partial z}+\frac{\partial w}{\partial x}\\ \frac{\partial u}{\partial y}+\frac{\partial v}{\partial x} & 2\frac{\partial v}{\partial y} & \frac{\partial v}{\partial z}+\frac{\partial w}{\partial y}\\ \frac{\partial u}{\partial z}+\frac{\partial w}{\partial x} & \frac{\partial v}{\partial z}+\frac{\partial w}{\partial y} & 2\frac{\partial w}{\partial z} \end{array}\right]$$

where $\bar{\bar{D}}=0.5\cdot \left[\left(\nabla \vec{V}\right)+{\left(\nabla \vec{V}\right)}^{T}\right]$ is the shear rate tensor. The stress on the wall, its normal component, and its two tangential components can be written as, respectively:

(A8) $$\vec{\tau }=\bar{\bar{\tau }}\cdot n,\tau_{n}=n\cdot \bar{\bar{\tau }}\cdot n,\tau_{t_{1}}=t_{1}\cdot \bar{\bar{\tau }}\cdot n\;\text{and}\;\tau_{t_{2}}=t_{2}\cdot \bar{\bar{\tau }}\cdot n$$

where **n**,***t***_1_, and ***t***_2_ are the unit vector in the normal and two tangential directions, respectively. The shear component of $\vec{\tau }$ has the vector form:

(A9) $${\vec{\tau }}_{\text{shear}}=\vec{\tau }-\left(\vec{\tau }\cdot n\right)n$$

Equation [A9] is used to calculate WSS with a magnitude: $|{\vec{\tau }}_{\mathrm{shear}}|=\sqrt{\vec{\tau }\cdot \vec{\tau }-{\left(\vec{\tau }\cdot n\right)}^{2}}$. The TAWSS can be written as follows:

(A10) $$TAWSS=\frac{1}{T}\int_{0}^{T}|{\vec{\tau }}_{\text{shear}}|\cdot dt$$

The OSI can be written as follows:

(A11) $$\text{OSI}=\frac{1}{2}\left(1-\frac{|\frac{1}{T}\int_{0}^{T}{\vec{\tau }}_{\text{shear}}\cdot dt|}{\frac{1}{T}\int_{0}^{T}\left|{\vec{\tau }}_{\text{shear}}\right|\cdot dt}\right)$$

The TAWSSG can be written as follows:

(A12) $$\text{TAWSSG}=\frac{1}{T}\int_{0}^{T}{\left[\frac{\partial \tau_{n}}{\partial n}+\frac{\partial \tau_{t_{1}}}{\partial t_{1}}+\frac{\partial \tau_{t_{2}}}{\partial t_{2}}\right]}^{1/2}\cdot dt$$

The transWSS can be written as follows:

(A13) $$\text{transWSS}=\frac{1}{T}\int_{0}^{T}\left|{\vec{\tau }}_{\mathrm{shear}}\cdot \left(n\times \frac{\int_{0}^{T}{\vec{\tau }}_{\mathrm{shear}}\cdot dt}{\left|\int_{0}^{T}{\vec{\tau }}_{\mathrm{shear}}\cdot dt\right|}\right)\right|\cdot dt$$

Equations [A10‐A13] were used to calculate the TAWSS, OSI, TAWSSG, and transWSS.

### Nomenclature

1. **SAR‐TAWSS at the anastomosis**: surface area ratio of low TAWSS $\left(=\frac{\text{Surface}\;{\text{area}}_{\mathrm{TAWSS}\leq 4\;\mathrm{dynes}{\mathrm{cm}}^{-2}}}{\text{Anastomotic surface area}}\times 100\%\right)$at the anastomosis between the graft and coronary artery. The end‐to‐side ‘anastomotic surface area’ is defined as the surface area of the proximal graft (5 mm length) to the anastomotic center plus the surface area of the distal coronary artery (5 mm length) to the anastomotic center. Surface area of TAWSS ≤ 4 dynes/cm^2^ refers to the disease‐prone site (Kleinstreuer et al. 2001; Malek et al. 1999).
2. **SAR‐TAWSS in the graft**: surface area ratio of low TAWS $\left(=\frac{\text{Surface}\;{\text{area}}_{\mathrm{TAWSS}\leq 4\;\mathrm{dynes}{\mathrm{cm}}^{-2}}}{\text{Surface area of the graft}}\times 100\%\right)$ in the graft. ‘Surface area of the graft’ refers to the surface area of the graft except for the anastomotic region.
3. **SAR‐OSI at the anastomosis**: surface area ratio of high OSI $\left(=\frac{\text{Surface}\;{\text{area}}_{\mathrm{OSI}\geq 0.15}}{\text{Anastomotic surface area}}\times 100\%\right)$at the anastomosis between the graft and coronary artery. Surface area of OSI ≥ 0.15 refers to the disease‐prone site (Huo et al. 2013; Nordgaard et al. 2010).
4. **SAR‐OSI in the graft**: surface area ratio of high $\left(=\frac{\text{Surface}\;{\text{area}}_{\mathrm{OSI}\geq 0.15}}{\text{Surface area of the graft}}\times 100\%\right)$ OSI in the graft.
5. **SAR‐TAWSSG at the anastomosis**: surface area ratio of high TAWSSG $\left(=\frac{\text{Surface}\;{\text{area}}_{\mathrm{TAWSSG}\geq 500\;\mathrm{dynes}{\mathrm{cm}}^{-3}}}{\text{Surface area of the graft}}\times 100\%\right)$ at the anastomosis between the graft and coronary artery. Surface area of TAWSSG ≥ 500 dynes/cm^3^ refers to the disease‐prone site (Fan et al. 2016).
6. **SAR‐TAWSSG in the graft**: surface area ratio of high TAWSSG $\left(=\frac{\text{Surface}\;{\text{area}}_{\mathrm{TAWSSG}\geq 500\;\mathrm{dynes}{\mathrm{cm}}^{-3}}}{\text{Surface area of the graft}}\times 100\%\right)$ in the graft.
7. **SAR‐transWSS at the anastomosis**: surface area ratio of high transWSS $\left(=\frac{\text{Surface}\;{\text{area}}_{\mathrm{trans}\mathrm{WSS}\geq 6\;\mathrm{dynes}{\mathrm{cm}}^{-2}}}{\text{Anastomotic surface area}}\times 100\%\right)$ at the anastomosis between the graft and coronary artery. Surface area of transWSS ≥ 6 dynes/cm^2^ refers to the disease‐prone site (Fan et al. 2016).
8. **SAR‐transWSS in the graft**: surface area ratio of high transWSS $\left(=\frac{\text{Surface}\;{\text{area}}_{\mathrm{trans}\mathrm{WSS}\leq 6\;\mathrm{dynes}{\mathrm{cm}}^{-2}}}{\text{Surface area of the graft}}\times 100\%\right)$in the graft.
